# The Use of Religious Capital as a Coping Strategy in Self-care by Type 2 Diabetes Patients in a Ghanaian Hospital

**DOI:** 10.1007/s10943-022-01722-2

**Published:** 2022-12-22

**Authors:** Kwadwo Ameyaw Korsah

**Affiliations:** Department of Adult Health, School of Nursing and Midwifery, College of Health Sciences, University of Ghana, P. O. Box LG43, Legon, Accra, Ghana

**Keywords:** Religiosity, Spirituality, Self-care, Type 2 diabetes, Qualitative research, Ghana

## Abstract

Given the importance Ghanaians attribute to spirituality and religiosity in terms of disease causation and management, this study explored the use of religious capital as a coping strategy by individuals with type 2 diabetes mellitus in self-care at the Techiman Holy Family Hospital Diabetes Clinic in the Bono East Region of Ghana. An exploratory descriptive qualitative research design was employed for the study. Semi-structured interviews were conducted with a convenience sample of twenty-seven (27) individuals recruited from the diabetes clinic. Content analysis was employed to find themes, which included: (1) Use of Prayer and Fasting for Courage from God, (2) Reliance on God as the Creator of Human Beings who Cures and Heals Diseases in the Body, (3) God as Source of life in times of Illness (Drawing life from God in times of illness), (4) Faith and Hope in God, and (5) Doctors and Nurses as Substitutes for God. The findings advocate the need to incorporate religiosity and spirituality into the provision of healthcare for individuals with diabetes to help them live productive lives.

## Introduction

Religious capital is defined as the religious resources available to individuals and groups through their affiliation with a particular religion, which are employed to cope with an array of harsh events, illness and problems (Finke, 2003; Vasconcelos, 2017). The religious resources include but are not limited to, beliefs, shared values, practices as well as the support from religious groups. It is believed that religion and/or spirituality has a positive influence on health and wellness outcomes of individuals (Koenig, 2012). A number of research studies such as Parson et al. (2006), Curlin et al. (2007) and Koenig, (2008) have noted that there is an association between spirituality, religion and physical health in addition to mental health. Additionally, Parson et al. (2006) and Koenig (2012) have indicated the importance of religion/religious practices and spiritual beliefs and have established a correlation between religious or spiritual activities/practices such church attendance, religious affiliation and membership to be beneficial for individuals with chronic conditions such as diabetes, cardiovascular conditions, hypertension, stroke, cancers and other general health conditions. Curlin et al. (2007) found that religion/spirituality was valued significantly by patients in their interactions with primary care doctors. Existing literature has explored several mechanisms by which religion and spirituality may influence health outcomes. For instance, Bozek et al. (2020) and Kor et al. (2019) studied psych-dynamic processes, health behaviors and practices, psychosocial effects and God and transcendental power in health and illness.

Onyishi et al. (2021), Yuniarti et al. (2013) and Kanbara et al. (2008) posit that religiosity and spirituality are employed by patients with diabetes mellitus to cope with emotional stressors such as anxiety, depression and apprehension. In a larger and more specific contexts, communities and societies resort to spiritual approaches in practical ways to manage illness and health situations (Abdoli et al., 2011; Casarez et al., 2010; Chew et al., 2011; Villani et al., 2019).

### Background

MURRAY and Agyare (2018) have argued that Ghanaians appear to be religious in nature. There is immediate tendency to relate almost every occurrence, such as diseases, bad demeanors of individuals and even outcomes of political elections to spirituality. According to the global ranking of highly religious nations, Ghana was among the top 20 most religious nations (Smith, 2018). The report indicated that about 95% of Ghanaians are very religious. The importance Ghanaians ascribe to spirituality and religiosity in disease causation and management (Assimeng, 2010; Murray & Agyare, 2018; Twumasi, 2005) and their general outlook on spirituality and religiosity in many situations (Assimeng, 2010; Twumasi, 2005) have been interrogated quite extensively. In this exploration, the extent and effectiveness of the use of religious capital as a coping strategy by individuals with type 2 diabetes mellitus in self-care is investigated.

The International Diabetes Federation report in (2014) indicated that diabetes affects 6.3% of the Ghanaian population with type 2 diabetes accounting for 90–95% of all cases of diabetes mellitus. Within the Ghanaian context, type 2 diabetes appears to be associated with increasing age and obesity, and it is also prevalent in the urban settings compared with the rural areas (Asamoah-Boaheng et al., 2019). There exists a plethora of research on the significance of religion and spirituality and their links to self-care among Ghanaians with diabetes mellitus. Assimeng (2010) has documented some perceived beliefs held by individuals with diabetes. According to him, there is belief that the condition is caused by spiritual forces. Spiritual activities like prayers, use of talisman, invocations, dependence on deities and God are perceived to be the main treatment for diabetes mellitus (Danquah et al., 2012 and Assimeng, 2010). These perceived beliefs about the causes and treatment for diabetes mellitus offer healthcare providers a better understanding of how to give patients culturally sensitive care.

### Significance of this Study

This research is aimed at finding the extent of the use of religious capital in the enactment of coping method in self-care among individuals with type 2 diabetes mellitus. The research questions which guided the study are: **(**1). What is the extent of the use of religious capital as a coping strategy in self-care among individuals with type 2 diabetes mellitus? (2). How do individuals with type 2 diabetes benefit from the use of religious capital in self-care?

The findings of this study may help to understand the association between the use of religious capital as a coping strategy in people with type 2 diabetes mellitus and its overall effects on diabetes outcome. They may further help to understand how people seek meaning in religious and spiritual beliefs and how that is beneficial in diabetes self-care. Furthermore, they may give a better understanding of how to integrate spirituality into programs of faith-based groups in supporting individuals living with chronic conditions and diabetes in particular. Finally, the findings may be used by patient advocacy groups and healthcare institutions as a reference material on how to support patients spiritually in difficult situations.

## Literature

### Influence of Religious Beliefs on Health-Seeking Behavior of Individuals

Beliefs about health and illness are culturally and socially constructed, and they often influence health-seeking behaviors of people and their decisions about treatment of a condition (Kahissay et al., 2017; Wallin & Ahlstrom, 2010). Consequently, the belief system to a large extent is likely to influence how people may experience and construe self-management (Lim et al., 2012; Sowattanangoon et al., 2009). Studies demonstrate that religious belief such as faith in God has helped in promoting the health of individuals with chronic conditions such as rheumatoid arthritis and systemic sclerosis (Kobayashi et al., 2015; Svensson et al., 2020). Other studies have noted that spiritual beliefs are vital in the management of individuals with diabetes mellitus and other chronic health conditions (Onyishi et al., 2021) and beyond management. Spirituality has also been cited to impact quality of life, quality of care, as well as satisfaction of patients with diabetes mellitus (Meichenbaum, 2008). This is because spiritual beliefs and activities may assist individuals with diabetes and other chronic diseases in coping by providing support, self-assurance as well as hopefulness (Newlin et al., 2008). However, other studies have also indicated that religious belief may serve as a barrier in self-care among patients with chronic illness such as diabetes (Bai et al., 2009; Hushie, 2019). As expected, religious beliefs may interfere with the coping resources, particularly when the patient neglects self-care activities and depends exclusively on prayers, meditations, holy rites and devotions to manage the health condition (Newlin et al., 2008).

### Religious Practices and Coping with Diseases

SAMUEL-HODGE et al. (2000) and Casarex et al. (2010) have suggested how individuals make sense of their relationships with God in managing their disease conditions. These interactions with God are established through religious practices such as prayers, listening to gospel music, words of faith and hope on radio and reading religious passages from gospel literature (Kilbourne et al., 2009; Casarex et al., 2010). In other studies, God is regarded by patients as the source of their illnesses and also patients rely on God for full recovery, empowerment and freedom (Abdoli et al., 2011; Peprah et al., 2018; Yuniarti et al., 2013). For some patients, God is seen as their source of knowledge in managing the condition (Peprah et al., 2018). Patients also perceive that God works through healthcare professionals to heal their diseases (Peprah et al., 2018). Additionally, God is seen as a collaborative care giver who works with both the patients and healthcare providers for the sake of the patients’ recovery (Polzer & Miles, 2007; Casarex et al., 2010). Patients also believe that there is the need to surrender self-care management of chronic diseases such as diabetes to the will of God (Polzer & Miles, 2007). In other words, it is necessary for patients to wait for God to heal their disease conditions at his own pace and time (Polzer & Miles, 2007; Casarex et al., 2010). The submissive perspective of patients in relinquishing to the will of God to heal the condition seems to be fatalistic in nature since it may serve as a barrier to self-care in illness situation and adherence to treatment in general (Bai et al., 2009; Wallin & Alhstrom, 2010). A study on spiritual coping in 41 countries around the globe found that most of the respondents acknowledged spiritual care as an essential component of palliative care in which most patients used their reliance on transcendence of God for hope and survival (Ramondetta et al., 2013). Stewart et al. (2011) also recounted positive association between religiosity and coping with glaucoma in diabetes patients.

Coping strategies are about the use of any effort intended for “a problem management and emotional regulation, which may give rise to outcomes of the coping process such as psychological well-being, functional status, and adherence” (Glanz & Schwarts, 2008, p. 213).

## Methods

### Research Design

An exploratory descriptive qualitative research design was chosen with the aim of exploring and describing spiritual coping approaches of individuals with type 2 diabetes (Shields & Rangarjan, 2013).

### Sample and Sampling Technique

#### Recruitment of Research Participants

All 42 participants were recruited at the diabetes patients’ clinic through convenience sampling method. Names of the individuals with diabetes were obtained through the patients’ attendance register at the Techiman Holy Family Hospital Diabetes Clinic, located in the Bono East Region of Ghana. The participants were contacted and duly informed about the research process on one-on-one basis. Information sheet was given to each participant for more information about the study. Those who agreed to take part in the study were invited to sign a consent form. Arrangements were made for an audio-taped interview at the convenience of the research participant as well as the researcher.

#### Inclusion and Exclusion Criteria

Inclusion criteria for this study considered individuals newly diagnosed with type 2 diabetes mellitus by a medical doctor within 3 months with no experiences of comorbidities. The rational for this sample was to engage with diagnosed individuals who would easily remember their coping experiences, as such experiences would still be fresh in their minds. For these newly diagnosed individuals with type 2 diabetes, it was necessary to explore their religious coping experiences in order to offer them support at the early stage of the condition. Exclusion criteria took note of patients who were severely ill, and the diagnosis of type 2 diabetes mellitus had not been confirmed by a doctor.

### Data Collection

#### Interview Guide

Data were collected between July 1 and December 31, 2009, through the use of semi-structured interview guide prepared in English. Data collected in the local language, Twi, were later translated into the English language. The semi-structured interview guide consisted of two main sections. The first section was used to collect data on socio-demographic information of the participants and was planned to establish rapport with the research participants to obtain reliable data for the study. The second section centered on questions related to religious coping with diabetes. Questions under this session included: “From the time you were diagnosed as having diabetes mellitus, how have you been dealing with the condition based on your religious faith or belief?” Interview conversations with each research participant were audio-recorded and lasted between 45 and 60 min. Data collection and analysis proceeded at the same time.

#### Saturation of Data Collection

Initial analysis of the data and the themes that emerged helped shape the subsequent sampling and data. Though forty-two (42) participants were recruited to take part in the interviews, saturation of data occurred when the 27th participant was interviewed and no new information was forthcoming (Babbie, 2007; Bengtsson, 2016). All the twenty-seven (27) interviews were conducted in a designated office in the diabetes clinic for such exercises. Indeed, the location ensured enough privacy and unnecessary interruptions during the interviews.

### Data Analysis

Simple content analysis was employed to identify appropriate themes from the data. This was done after reading through the transcribed data from the 27 participants who were interviewed before saturation occurred (Bengtsson, 2016). Sense of ideas, words, meanings, statements, sentences, phrases and thoughts around the phenomenon under investigation were noted and documented. Subsequently, specific files were created containing common ideas, words, meanings, statements, sentences and thoughts around the phenomenon being studied. Specific names were then assigned to these files (Bengtsson, 2016). The primary files initially created were re-examined twice to bring common and similar ideas, statements, phrases, sentences, meanings, specific words together to identify the basic themes and sub-themes (Elo et al., 2014). As stated earlier, content analysis was employed for the data analysis to identify associations in the text and to bring to light essential links to the phenomenon under examination (Elo et al., 2014). Data collection and analysis took place concurrently, and four main themes and sub-themes were identified.

### Ethical Considerations

Scientific and ethical approval of the research was attained from the Faculty of Health and Life Sciences (HLS) Research Ethics Committee of De Montfort University, Leicester, in the UK (Reference 347-04/23/08). Permission to carry out the research was also granted by the local Hospital Management Team (HMT) of the Techiman Holy Family Hospital (HFH) and the Techiman Municipal Health Directorate (MHD) in Ghana. Local members of the National Diabetes Association in Techiman municipality, Ghana, also gave permission for this research to be conducted among its members in the hospital. Research participants who decided to participate in the research were made to sign a consent form prior to the interviews, assured of anonymity and confidentiality and informed of their right to leave the study at any point without being chastised. Pseudonyms were given to the research participants to conceal their true identity. Biographic data of the research participants were detached from the data collected to avoid connections between them.

### Trustworthiness of the Research Process

Trustworthiness of this study centered on three (3) key approaches. Firstly, efforts were made to conduct extensive interviews with the participants for sufficient and reliable information regarding the subject matter. Secondly, two (2) extended family members of the author diagnosed with type 2 diabetes mellitus at the time of this study were invited to participate in the piloting of the interview guide. This helped to refine the ultimate version of the interview guide. Thirdly, individual research participants were granted the opportunity to decide together with the researcher, the location and time for the interview. This ensured that the research participants voluntarily take part in the research interviews without being coerced.

### Findings

#### Socio-Demographic Information

Twenty-seven (27) individuals newly diagnosed with type 2 diabetes mellitus were interviewed. The ages of the participants ranged from 38 to 63 years, with 15 males and 12 females. Out of the 27 participants, 26 were Christians and 1 Muslim (Table 1).

**Table 1:** Demographic characteristics of the participants (*n* = 27)

Participants	Age	Gender	Religion
Participant 1	48	Male	Christian
Participant 2	52	Female	Christian
Participant 3	63	Male	Muslim
Participant 4	50	Female	Christian
Participant 5	46	Female	Christian
Participant 6	60	Female	Christian
Participant 7	62	Male	Christian
Participant 8	39	Female	Christian
Participant 9	40	Female	Christian
Participant 10	55	Male	Christian
Participant 11	45	Female	Christian
Participant 12	38	Male	Christian
Participant 13	45	Male	Christian
Participant 14	50	Male	Christian
Participant 15	49	Female	Christian
Participant 16	61	Female	Christian
Participant 17	62	Male	Christian
Participant 18	56	Male	Christian
Participant 19	59	Female	Christian
Participant 20	49	Female	Christian
Participant 21	44	Male	Christian
Participant 22	42	Male	Christian
Participant 23	62	Male	Christian
Participant 24	59	Female	Christian
Participant 25	39	Male	Christian
Participant 26	51	Male	Christian
Participant 27	53	Male	Christian

#### Religious/spiritual coping strategies

Religious capital as a coping strategy among patients with type 2 diabetes centered on 5 core themes (Table 2) which were utilized in the face of diabetes-related distress and other challenges confronting them following diagnosis. These included: (1) Use of Prayer and Fasting for Courage from God, (2) Reliance on God as the Creator of Human Beings who Cures and Heals Diseases in the Body, (3) God as Source of life in times of Illness—(Drawing life from God in times of illness), (4) Faith and Hope in God, and (5) Doctors and Nurses as Surrogates for God.

1. Use of Prayer and Fasting for Courage from God

**Table 2:** Five key themes/findings of religious coping strategies

No.	Theme
1	Use of prayer and fasting for courage from god
2	Reliance on god as the creator of human beings who cures and heals diseases in the body
3	God as Source of life in times of Illness—(Drawing life from God in times of illness)
4	Faith and hope in god
5	Doctors and nurses as surrogates for god

The participants recounted their experiences on how prayers as a form of communication helped them to inform God of their diagnosis and the challenges thereafter, through which courage was gathered from God to deal with the condition perceived to be chronic in nature. A participant had this to say:

I know with prayers all things are possible, God heals when you communicate your problems to him in prayers. So, if you are not well and you are suffering from any condition like I am suffering from diabetes, you need to tell Him for courage in order to manage the condition (P12).

Similarly, another participant cited the need to pray in addition to fasting in order to communicate with God in times of trouble such as when one is sick, for God’s strength and power.

Through fasting and prayers, one is able to communicate with God and inform Him about what is happening to you for strength and power as well as directions, so it is all about fasting and prayers which will help in almost all cases especially when you have any disturbing condition (P5).

It is apparent to observe among these patients that they rely on prayers and fasting as a way by which their communication with transcendence offers them hope and courage to manage and deal with diabetes mellitus.

2. Reliance on God as the Creator of Human Beings who Cures and Heals Diseases in the Body

Some of the participants were also with the view that God creates human beings and viewed God as the architect of human life and therefore the source or origin of humans, who knows the beginning and the end of humans. This analogy seems to suggest that as the Supreme Being, God knows the origins of all diseases and therefore He is the Healer. The following are some of the exemplars from the participants:

As a Creator of human beings, God knows where diseases come from, so he has the cure for every disease, so I am sure that God will heal my diabetes (P10).

As a Master Architect of persons, the Creator can cure all diseases irrespective of its origin and how it is formed (P17).

Another participant who was emphatic about the nature of God in curing people with diabetes narrated thus:

Diabetes is nothing before God, so my condition will be healed by Him. It is about faith in God that all things are possible (P23).

The research participants were convinced that there is a cure for diabetes by virtue of their belief and reliance on God.

3. God as Source of Life in Times of Illness—(Drawing Life from God in Times of illness)

Other participants also viewed God as a source of life in dealing with their condition. They were of the belief that although the diabetes had developed, the effects of the condition may not be fatalistic considering their hope of life in God. To them, diabetes is a debilitating condition, but with the presence of God in their lives, the condition could be overcome. Some of their narratives include:

Yes, I know that diabetes is a very dangerous condition, however with the presence of God in my life, the condition has no effect on me. God is the source of life so it is the hope you have in him which will make you survive, because He is the Source of Life in times of diseases even if you are dying (P11).

By Divine’s power, diabetes has been overcome and so it is all about believing in God. The blood sugar levels can go down, even with prayers and having faith in him. Whatever you do will work for you and the condition will not harm you (P13).

From the patients’ narratives we gather the theme that participants believe with the power of God, blood sugar levels can reduce and there may not be any complications which can ultimately lead to death.

4. Faith and Hope in God

With faith and hope in the Supreme Being, participants in this study had inner peace and sense of autonomy from the hitches of living with a lingering condition like diabetes mellitus. They had assurance of confidence and optimism in God on matters related to the challenges with diabetes management. Some participants had this to say in relation to the faith and hope in the Supreme Being regarding treatment of the condition.

With faith and hope in God, any medication given by our care providers will work. If you don’t have hope and faith even the very powerful drug will not work for you, so it is when you have faith and hope that you are healed even if the drug is not strong enough (P27).

Having hope and faith is everything, not only in disease situation. It is everywhere, so with hope and faith in God, the disease is cured completely whether you are on medication or not. It is very important to consider that as part of any drug one is taking. You have to pray and with faith and hope you get healed (P15).

It is understandable from the participants’ stories that hope and faith in God is functional in relation to managing a chronic condition like diabetes mellitus with or without medicines.

5. Doctors and Nurses as Surrogates for God

The participants had the impression that although God heals in miraculous ways to cure diabetes, doctors and nurses also work to heal patients in place of God. Some of the participants articulated the following:

God is there to cure disease conditions of people, but He has also given the power and knowledge to doctors and nurses to help patients with any condition like diabetes. God will not be there physically for you to see him but through nurse and doctors and other people you get healed of your condition (P6).

God works through other people such as nurses and doctors to treat patients in many different ways. It may be an advice that a nurse or doctor can give you or by prescribing a medication for you but remember that God is the source of all knowledge, so He heals people through medical professionals and other people (P19).

From the participants’ exemplars, nurses and doctors and other healthcare professionals are surrogates of God in many situations in terms of disease management. God works through them for the benefit of all patients including persons living with diabetes.

## Discussion of Findings

### Perceived Beliefs in God for Managing Diabetes

Findings of this study centered on the perceived belief in God by the participants to help manage diabetes through ways such as prayers and fasting for courage from God. Fasting and prayers were perceived as a way of communicating with God. It is noted that the exact mechanisms by which religion and spirituality play in dealing with diseases are not well understood, yet there are studies such as Koffman et al. (2008) who have noted that religion and dependence on God helps cancer patients in reducing the psychological and physical outcomes of illness. Similarly religious values as well as beliefs may have crucial effects on the life of individuals. For instance, beliefs and religious teachings may have the power to let individuals “interpersonally” deal or manage their own “temperament, annoyance and anger” physiologically, cognitively and psychologically (Rosemarin & Koenig, 2020). Religious beliefs may therefore be utilized to support oneself in challenging circumstances (Khalsa & Newberg, 2021; Monjezi et al., 2012). This assertion may be subjective, and much of an individuals’ behavior may depend rather on the doctrines and teachings of one’s faith. The analogy may be of value in the discussions of the efficacy of religious beliefs, faith, meditations as well as prayers in the management of chronic diseases such as diabetes mellitus. The religious doctrines and teachings may rather influence individuals’ behavior in self-management of diabetes and similar conditions, if the doctrines of that particular religious group are included in the health promotion of members of the group. So, the mere belief in God and Divine power and the use of prayer or fasting and meditation may not have any meaningful effect on the disease outcome.

### Religiosity in Diabetes Self-care and Glycemic Control

The findings also indicate that participants in this study coped spiritually by relying on God as a creator of human beings who cures all diseases in the body including diabetes mellitus. As already acknowledged in a study by Watkins et al. (2013), the Divine power is able to heal all diseases and also be a source of encouragement and stimulation for diabetes patients in self-care management, but as to how this works to bring blood glucose within target range remains an unanswered question. Research findings by Chew Boon How et al. (2011) also seem to offer some explanation to the extent to which spirituality helps diabetes patients in managing or coping with the high glycemic levels. They posit that glycemic control may be indirectly related to individual’s religious orientation, religious practices, beliefs in general and faith which seem to shape or modify person’s lifestyle in a positive manner which ultimately may be manifested in better outcomes of the diabetes in terms of glycated hemoglobin (HbA1c) and limited or devoid of complications.

### Patients’ Sense of Relationships with God in Healing Diabetes

The research participants also perceived the nurses and doctors as surrogates of God. There was belief that God through nurses and doctors, heals patients with diabetes mellitus. Faith, hope, knowledge and power of healing from the nurses and doctors were equated with God’s power by the diabetes patients. There are additional ways by which other patients adopt to make sense of their affiliations and relationships with God in order to manage their disease conditions and other problems or challenges. For instance, three categories were identified by Permana (2018) on how patients make sense of their everyday interactions and relationships with God. These included “supportive, collaborative and submissive” (Permana, 2018, p. 190) relationships. On the supportive relationship, some patients regard God as caring and helpful in nature and as the foundation of their health and illness state (Abdoli et al., 2011). The supportive relationship with God may also augment self-confidence, self-determination and enhance self-management (Casarez et al., 2010). Additionally, the supportive relationship with God may empower patients to be independent in controlling and regulating the illness (Abdoli et al., 2011; NHS, 2020; Polzer & Miles, 2007). The God-person collaborative relationship in self-care management focuses on the shared responsibilities of the patient and God in dealing with the condition. The patients being chief actors in their own care, and God offering the needed support in this care (Casarez et al., 2010; Polzer & Miles, 2007). This may seem to be an “active personal exchange with God” (Permana, 2018, p. 190) and “a self-incorporated form of religion” (Pargament et al., 1988, p. 90). The coping through submissive act by the patient refers to the patient “relinquishing self-care and management” of the condition to God’s will and power (Permana, 2018; Polzer & Miles, 2007) by waiting for the Supreme God to cure or heal the disease condition in His own time (Permana, 2018; Polzer & Miles, 2007). This may be a fatalistic perception of the disease condition by the patient which has the tendency to hamper complete self-caring efforts of the patient in all facets of illness management of diabetes patients. For instance, the submissive act of the patient may hamper medication, diet and exercise adherence as the patient may not put in any efforts to deal with the condition but to wait for God’s intervention. However, Permana, (2018); Lazarus and Folkman (1984) argue that the fatalistic nature of submission to the will of God in disease management does not necessarily pose any danger to the patient. Rather, it offers the patient inner strength, peace and harmony to deal with the condition. The perception of submission to God in self-care is also evident in Chew Boon How et al. (2011, p. 26) where Moslems believe in submitting to the will of Allah and, thus, they may not take as much care of their glycemic control but may leave it to “fate” or “destiny.” Similarly, some Sudanese also perceive diabetes mellitus as a form of punishment from mystical powers such as witches or as an esteem and love from God; as a test of their trust and faith in God (Ahmed, 2003); or as a will of God (Yuniarti et al., 2013). The findings of this study on the use of religious capital as coping strategy are in line with the findings reported by Monjezi et al., 2012, Permana, 2018; Jafari et al., 2014; Bredle et al., 2011; De Graft Aikins, 2005; Yuniarti et al., 2013. It is worth noting that the import of the style of religious coping in all these studies rests on the relevance of faith, belief systems, the trust and hope in God to deal with disease conditions irrespective of the type of the condition. There exists an array of religious coping styles employed by patients including individuals diagnosed as having diabetes mellitus, but the choice of a particular strategy appears to depend on individual’s perceived religious beliefs and cultural orientation.

### Religious Coping and Barriers to Diabetes Self-care

Other findings of religious and spiritual coping by the type 2 diabetes centered on the fact that God is the source of life and patients have the hope that they would survive despite the debilitating effects and chronicity of diabetes. This is further corroborated by Duke (2021) and Bredle et al. (2011) who categorized the effectiveness of spiritual coping in three main areas as having peace, meaning and faith in God for healing and recovery from diabetes. Jafari et al. (2014) also identified that “higher meaning and peace in the Ultimate Being were related to better physical, social, emotional, and functional well-being as well as quality of life, whereas higher faith was only associated with physical well-being” (Jafari et al., 2014, p. 3). Nevertheless, religious faith and trust in God for healing may hamper self-care practices of patients and also create barriers in adaptation (Yannez, 2009). For instance, spiritual practices and beliefs and faith which may serve as a barrier to adjustment may be linked to a negative perception and coping by patients. Within this circle, diabetes patients may assume that the ill-health condition experienced is a punishment from God due to their bad demeanors. Such patients in an attempt to cope and adjust with the condition may end up in prayer camps and other places where they are often expected to engage in fasting, a treatment mode which may not be an appropriate form of treatment. Some patients may later report to the hospital in deteriorated diabetes state (De Graft Aikins, 2005).

### Implications for Practice

The findings of this study suggest the need for healthcare providers such as nurses, doctors and hospital-based pastoral teams to integrate religiosity and spirituality into health and nursing care of patients. This may help the patient to live with chronic diseases and diabetes in particular. This may be done when the spiritual needs of their patients are factored into their care. This could be exemplified when doctors, nurses as well as chaplaincy teams in the course of their routine roles in patient care pray together with them, offer hope and advocate for reliance on God in illness situations. Additionally, health institutions may incorporate faith-based approaches in patient care whereby patients’ spiritual upliftment may be enhanced through singing and praises to God at specific times. In some cases, spiritual retreats when organized for patients in the hospital could tailor the preaching of God’s words to expatiate the need for patients to have faith in God for healing of their disease conditions. These findings highlight the relevance of healthcare providers recognizing how people seek meaning in religious and spiritual beliefs in ill-health situations.

### Study Limitations

The researcher is a professional nurse who has worked in this role for 20 years. This exposes the findings of this study to bias. The researcher tried to reduce his biases by bracketing his suppositions. This was achieved by asking the research participants simple open-ended and flexible questions which allowed the research participants to give thorough narratives of their experiences on the subject matter. With the data saturation occurring on the 27th participant and with this limited number of participants, it may be difficult to generalize the findings. However, to ensure generalization of findings, it may be necessary to advance this study in manifold research sites. Data for this study were analyzed manually, which could result in methodological and operational errors as compared with computer software research data analysis which seem to be consistent and cost-effective (Banner & Albarran, 2009). Nevertheless, it is noted in qualitative research that certain gestures and behaviors exhibited by research participants may not be analyzed by the computer software. Opting to analyze the data manually enabled the study to include these gestures to compliment the nuanced analysis intended for this research.

## Conclusion

The article focused on religious and spiritual coping, the extent and its effectiveness among individuals with type 2 diabetes in Ghana. The patients employed diverse religious and spiritual coping mechanisms to manage the condition, and these include prayer and fasting for courage from God, dependence on God as the creator of human beings who cures and heals diseases, God as source of life in illness situations, having faith and hope in God, and the role of nurses and doctors as surrogates of God in diabetes self-care. The findings inform healthcare providers to consider religiosity and spirituality in particular in the care of individuals with diabetes mellitus. It is suggested that further research into religious and spiritual coping mechanisms and the belief in God would broaden understanding on how religion and spirituality contribute to the control of blood glucose levels in individuals with diabetes. Such an investigation may add value to the discussions.
